# Genetic factors associated with hidradenitis suppurativa, a literature review

**DOI:** 10.1097/JW9.0000000000000158

**Published:** 2024-06-14

**Authors:** Shannon M. Eble, Oliver J. Wisco, Luigi Boccuto, Brian Laffin, Veronica G. Parker, Nicole J. Davis, Heide S. Temples

**Affiliations:** a College of Behavioral, Social, and Health Sciences, College of Nursing, Clemson University, Clemson, South Carolina; b Bristol Myers Squibb, Princeton, New Jersey; c Department of Dermatology, The Warren Alpert Medical School, Brown University, Providence, Rhode Island

**Keywords:** acne inversa, genetics, hidradenitits suppurativa

## Abstract

**Background:**

Hidradenitis suppurativa (HS) is a chronic inflammatory disease characterized by deep-seated, painful lesions most frequently occurring in intertriginous areas of the skin. HS leads to poor quality of life in affected individuals and is difficult to diagnose and treat.

**Objective:**

Understanding the genetics associated with familial inheritance may lead to a better understanding of the pathogenesis of this debilitating disease.

**Methods:**

Articles published until March 9, 2023, were identified in PubMed using the following search terms: hidradenitis suppurativa and gene* or acne inversa and gene*.

**Results:**

The rate of monogenic mutations associated with HS is less than 7%, with the most common genetic mutations reported in sporadic and familial HS cases being in *NCSTN* and less frequently in *PSENEN.* Individuals with mutations in the gamma-secretase complex tended to have more severe HS and an early age of onset.

**Limitations:**

This study was limited to the case studies available in PubMed, the majority of which used targeted gene panels to detect genetic mutations.

**Conclusion:**

Approximately 30% of individuals diagnosed with HS report having a positive family history; however, very few studies demonstrate monogenic familial transmission of HS. The case studies of syndromic HS reported a variety of genetic mutations associated with HS, some of which were familial, while others were sporadic, suggesting that other pathways may be involved in the pathogenesis of HS and other potential mutations that have yet to be evaluated. More research is needed to understand the genetic mutations in HS.

**What is known about this subject in regard to women and their families?**

- Hidradenitis suppurativa (HS) is a chronic, painful inflammatory disease that disproportionately affects women.
- Approximately 30 to 40% of patients report having a family history of HS.
- Accurate diagnosis of HS can take up to 7 to 10 years.

**What is new from this article as messages for women and their families?**

- This article highlights the need for further genomic interrogation to better understand the familial risk factors associated with developing HS.
- Evaluating the distinct subtypes of HS—familial, sporadic, and syndromic—may lead to better genotype-phenotype correlations, allowing for better diagnostic tools and more effective therapeutic options.

## Introduction

Hidradenitis suppurativa (HS), or acne inversa is a chronic, painful inflammatory disease characterized by deep-seated, painful nodules, abscesses, and draining tunnels in the apocrine gland-bearing regions of the body.^1,2^ Until recently, HS was considered a rare disease; however, we now know that prevalence is at least 1% in the United States and is seen at variable rates across geographical regions, and the onset of the disease typically occurs after puberty.^3–6^ HS disproportionately affects African Americans in prevalence and severity and affects women to men at a 3:1 ratio.^4,7–10^ Individuals with HS often have a poor quality of life, and are often of a low socioeconomic status, with many patients unable to work due to the severity of their disease or having lower productivity than their peers.^11–15^

HS is diagnosed clinically and varies greatly in presentation and many scoring systems exist to characterize disease severity.^13^ Because disease presentation varies, there is often a significant delay from the onset of symptoms to disease diagnosis, approximately 7 to 10 years on average.^11,14^ HS therapeutic options are often based on disease severity and include topicals, biologics, and surgical options.^6,11^ HS is a disease with many data gaps in regard to knowledge of disease pathogenesis, effective treatment, as well as accurate and timely diagnosis.^15^

HS patients are suspected of having a genetic predisposition along with environmental or lifestyle factors contributing to the development of disease, with smoking and obesity being the most common.^1,2,4,16–18^ Approximately 30% of patients reported having a family history of HS and in a large-cohort twins study, the narrow-sense heritability of HS was 77%.^19^ Several studies have shown that gamma-secretase mutations may be associated with the pathogenesis of HS.^20,21^ The purpose of this targeted review is to focus on the genetic factors described in the literature associated with HS.

## Methods

Articles published until March 9, 2023, were identified in PubMed using the following search terms: *hidradenitis suppurativa* and gene* or *acne inversa* and gene*.

**Fig. 1.**
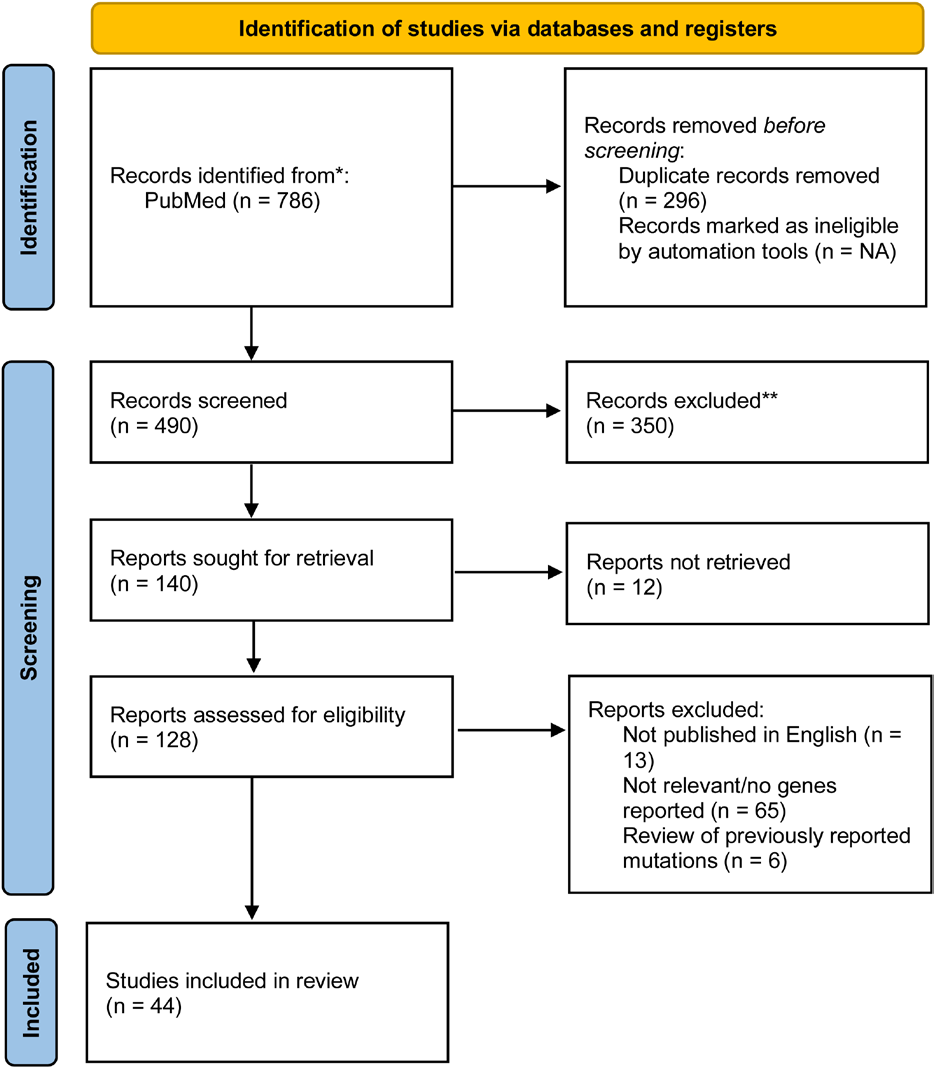
PRISMA flowchart of study selection.

Articles were included if they mentioned specific genetic mutations found to be present in individuals or families with HS or acne inversa and were published in English. This search yielded 786 articles and 296 duplicates were removed, leaving 490 articles that were assessed for eligibility. Articles that did not identify specific genetic mutations were excluded from the review, of which 44 studies met the inclusion criteria (Fig. 1).^22^ Literature reviews were excluded from this review; however, they were referenced for background information.

## Results

### Pathophysiology of HS

HS is a complex disorder involving both environmental factors, such as smoking, obesity, and hormones, as well as genetic factors; however, the exact pathophysiology is not well understood.^23^ A dysregulated innate and adaptive immune system has been observed in individuals with HS, with upregulation of proinflammatory cytokines and chemokines.^24–26^ There is still much to understand regarding the genetic factors associated with HS. Gamma secretase is important in the regulation of normal immune function and the maturation of hair follicle cells.^11^ Gamma-secretase complex (GSC) is a multisubunit, membrane-embedded, protease complex consisting of the following subunits: presenilin-1 (PSEN1) or presenilin-2 (PSEN2), presenilin enhancer gamma-secretase subunit (PSENEN), Nicastrin (NCSTN), anterior pharynx defective 1a or anterior pharynx defective 1b.^21^ GSC is involved in one of the cleavage steps that release Notch, which is required for activation of the Notch pathway. GSC and Notch are known to play an important role in the formation of epidermal cysts and comedones and dysregulation has been shown to increase the formation of these structures in HS patients.^21^ There have been many case studies reporting mutations in the GSC both in families with HS as well as sporadic cases.^21^ Additional case studies report HS in conjunction with other autoinflammatory disorders.^27^ Based on the review of the literature, there seem to be 3 distinct genetic classifications of HS: familial, sporadic, and syndromic.

### Genetic studies

In 1968, Knaysi et al.^28^ conducted a review of 45 subjects who underwent surgical intervention for the treatment of HS between 1932 and 1965 at Presbyterian Hospital, Columbia University, New York, and recorded a family history of HS in 3 of 18 subjects who were specifically asked, however, no additional information was provided. Fitzsimmons et al.^29^ evaluated the transmission history of HS in 21 English patients across 3 families in 1984 and was the first study to suggest transmission through a single gene with autosomal dominant inheritance. A year later the same group of researchers expanded the study to 62 HS patients across 23 families, where 11 families demonstrated transmission through several generations, consistent with single gene or Mendelian inheritance.^30^ As seen in the previous study, 3 of the families had a familial occurrence, while 9 of the families showed no family history at the time of the study, which the team noted may be due to inaccurate diagnosis and false negative family history.^30^ Fifteen years later, Von der Werth et al.^31^ set out to test the validity of the Fitzsimmons et al. study and reviewed the 14 surviving probands and their families, where they saw the same autosomal dominant inheritance pattern seen in the previous study in those where a positive family history had been noted previously.

### Familial HS

Approximately 30 to 40% of individuals with HS report having at least one affected first-degree relative, indicating genetics are likely involved in the pathogenesis.^11^ In 2006, Gao et al.^10^ mapped the candidate HS gene to chromosome 1p21.1–1q25.3 by genome-wide linkage analysis in a 4-generation Chinese family, though no specific gene was identified due to the large genomic region. Another study conducted a few years later evaluated 29 individuals from 2 large, multigenerational families with HS by linkage analysis with the 9 satellite markers used in the previous study.^5^ The results of this study ruled out 1p21.1–1p25.3 involvement in the pathogenesis of HS in the 2 families evaluated; however, HS presented as an autosomal dominant trait with 100% penetrance with no skipping of generations observed.^5^

Wang et al.^32^ was the first group to report mutations in the GSC in 6 Han Chinese families; families 1 and 2 had different *PSENEN* mutations, while families 3–6 had either *NCSTN* or *PSEN1* mutations. In families 3–6, reverse transcription polymerase chain reaction demonstrated a reduction in transcript expression in individuals with a frameshift or nonsense mutation in *PSEN1* or *NCSTN*, indicating these mutations ultimately lead to a loss of function, leading to defective Notch signaling.^32^ Twenty-five familial HS case studies were reviewed and revealed 37 unique mutations in the GSC (Table 1). Mutations in *NCSTN* were present in all affected family members of 31 families, while mutations in *PSENEN* were present in all the affected family members of 5 families and mutations in *PSEN1* in one family; mutations in these genes were not seen in the family members and control subjects without HS. The cases reviewed exhibited autosomal dominant inheritance with incomplete penetrance. This data indicates that mutations affecting GSC are involved in some familial HS cases; however, there are likely other genes involved in the molecular pathogenesis of HS that have not yet been identified.

**Table 1:** Genetic mutations in individuals with familial HS

Diagnosis	Gene	Change in DNA	Change in protein	Origin	Reference
HS	*NCSTN*	c.687insCC	p.Cys320ProfsX31	Indian	Ratnamala et al., 2016^33^
AI (HS)	*NCSTN*	c.477C>A	p.(C159X)	Chinese	Xiao et al., 2016^34^
AI (HS)	*NCSTN*	c.944C>T	p.A315V	Chinese	Zhang et al., 2016^35^
HS	*NCSTN*	c.751_752del	p.Leu251Valfs*2	NR	Zhang et al., 2022^36^
HS	*NCSTN*	c.671_682del	p.Val224Thr227del	NR	Mintoff et al., 2021^37^
HS	*NCSTN*	c.447delC	p.N150Ifs*52	Chinese	Liu et al., 2022^38^
HS	*NCSTN*	c.1285C>T	p.R429X	Japanese	Nishimori et al., 2020^39^
HS	*NCSTN*	c.1912_1915del	p.S638fs	Dutch	Vossen et al., 2020^40^
HS	*NCSTN*	c.97G>A	p.Gly33Arg	Japanese	Takeichi et al., 2020^41^
AI (HS)	*NCSTN*	c.793_794insA	p.Thr265AsnfsX8	Chinese	Xiao et al., 2020^42^
AI (HS)	*NCSTN*	c.751_752delCT	p.Leu251ValfsX2	Chinese	Xiao et al., 2020^42^
AI (HS)	*NCSTN*	c.218delC	p.P73Lfs*15	Chinese	Wu et al., 2018^43^
AI (HS)	*NCSTN*	c.617C>A	p.S206X	Chinese	Shi et al., 2018^44^
AI (HS)	*NCSTN*	c.1752delG	p.E584DfsX44	Han Chinese	Wang et al., 2010^32^
AI (HS)	*NCSTN*	c.1551 + 1G>A	p.A486_T517del	Han Chinese	Wang et al., 2010^32^
AI (HS)	*NCSTN*	c.349C>T	p.R117X	Han Chinese	Wang et al., 2010^32^
AI (HS)	*NCSTN*	c.450_459del	p.Ser151GInfsX48	Chinese	Wu et al., 2020^45^
HS	*NCSTN*	c.1101 + 1G>A	No variant transcript	British	Pink et al., 2011^46^
HS	*NCSTN*	c.1352 + 1G>A	NR	Chinese	Liu et al., 2011^47^
HS	*NCSTN*	c.210_211delAG	p.Thr70fsX18	Chinese	Liu et al., 2011^47^
HS	*NCSTN*	c.1695T>G	p.Y565X	Chinese	Li et al., 2011^9^
HS	*NCSTN*	c.632C>G	p.P211R	Chinese	Li et al., 2011^9^
HS	*NCSTN*	c.449C>T	p.R117X	White	Liu et al., 2016^48^
HS	*NCSTN*	c.1258C>T	p.Q420X	Chinese	Jiao et al., 2013^49^
HS	*NCSTN*	c.1702C>T	p.Q568X	Japanese	Nomura et al., 2013^50^
HS	*NCSTN*	c.647A>C	p.Q216P	Chinese	Zhang et al., 2013^51^
HS	*NCSTN*	c.233G>A	p.V75I	Chinese	Zhang et al., 2013^51^
HS	*NCSTN*	c.582 + 1delG	NR	Japanese	Nomura et al., 2013^50^
HS	*NCTSN*	c.1300C>T	p.Arg434X	French	Miskinyte et al., 2012^52^
HS	*NCTSN*	c.487delC	p.Gln163SerfsX39	French	Miskinyte et al., 2012^52^
HS	*NCTSN*	c.1768A>G	pSer590AlafsX3	French	Miskinyte et al., 2012^52^
AI (HS)	*PSEN1*	c.725delC	p.P242LfsX11	Han Chinese	Wang et al., 2010^32^
AI (HS)	*PSENEN*	c.13>T	p.R3X	Chinese	Qian et al., 2022^53^
AI (HS)	*PSENEN*	c.66delG	p.F23LfsX46	Han Chinese	Wang et al., 2010^32^
AI (HS)	*PSENEN*	c.279delC	p.F94SfsX51	Han Chinese	Wang et al., 2010^32^
HS	*PSENEN*	c.66_67insG	p.Phe23ValfsX98	British	Pink et al., 2011^46^
HS	*PSENEN*	c.60_66delG	p.F23LfxX10	Chinese	Chen et al., 2022^54^

AI, acne inversa; HS, hidradenitis suppurativa; NR, not reported.

### Sporadic HS

Genetic mutations present in sporadic cases of HS have been reported less frequently than in familial HS (Table 2). Pink et al.^55^ evaluated 48 individuals with HS recruited from a referral clinic for mutations in *NCTSN, PSEN1*, and *PSENEN* and subsequently identified 3 *NCTSN* variants, 2 consistent with the pathogenesis of HS in patients without a family history. The 2 individuals with the likely pathogenic variants of NCTSN both had severe disease, high body mass index, and were current smokers; one of the patients had an early age of onset, age 13, and the other patient was 35 at the age of onset.^55^ The results of this study suggest that genetic mutations of *NCTSN, PSEN1*, and *PSENEN* are present in less than 7% of all HS cases. Another study evaluated samples from 2 large, phase III clinical trials for the potential genetic association of *NCSTN*.^48^ Next-generation sequencing of the entire *NCSTN* gene was initially performed in samples from 95 subjects enrolled in either study and select variants found in these subjects were further evaluated in 443 subjects with HS.^48^ One missense *NCTSN* mutation was identified in a patient without a family history of HS.^48^ Liu et al.^56^ reported a 29-year-old male smoker with no family history of HS who had a deletion mutation in *PSENEN,* which was the first time a *PSENEN* mutation was reported in a sporadic case of HS. In another study, Vural et al.^21^ evaluated DNA from 38 individuals diagnosed with HS using Sanger sequencing to investigate the frequency of variants of GSC genes *APH1A, APH1B, PSENEN, NCSTN, PSEN1,* and *PSEN2*. This study revealed a pathogenic *NCSTN* mutation in one patient with sporadic HS.^21^ These studies demonstrate that mutations in the GSC occur in a minority of HS cases. Mutations in genes of the GSC may be associated with more severe HS and earlier age of onset in familial and sporadic HS.^21^ In severe cases of HS, as well as in subjects with early age of onset, genetic testing for GSC mutations may be warranted.^21^

**Table 2:** Genetic mutations in individuals with sporadic HS

Sex	Diagnosis	Gene	Change in DNA	Change in protein	Origin	Reference
M	AI (HS)	*NCSTN*	c.1555dupA	p.T519NfsX9	Chinese	Qian et al., 2022^53^
M	HS	*NCSTN*	c.2584_2585delCA	NR	Chinese	Xiao et al., 2018^57^
NR	HS	*NCSTN*	c.38delG	p.Gly13Glufs*16	German	Vural et al., 2021^21^
F	HS	*NCSTN*	c.553C>A	p.Asp185Asn	NR	Pink et al., 2012^55^
F	HS	*NCSTN*	c.996 + 7G>A	NR	NR	Pink et al., 2012^55^
M	HS	*NCSTN*	c.1101 + 10A>G	NR	NR	Pink et al., 2012^55^
F	HS	*NCSTN*	c.1229C>T	p.A410V	White	Liu et al., 2016^48^
M	AI (HS)	*PSENEN*	c.66delG	NR	Chinese	Liu et al., 2016^56^

AI, acne inversa; HS, hidradenitis suppurativa; NR, not reported.

### Syndromic HS

In a minority of cases, HS has been described in combination with other autoinflammatory, genetic, and immune-mediated conditions and is referred to as syndromic HS.^58^ The autoinflammatory conditions associated with syndromic HS include: pyoderma gangrenosum, acne, and suppurative hidradenitis (PASH); pyodermic arthritis, pyoderma gangrenosum, acne, and suppurative hidradenitis; psoriatic arthritis, pyoderma gangrenosum, acne, suppurative hidradenitis; pyoderma gangrenosum, acne, suppurative hidradenitis, and ankylosing spondylitis; and PASH overlapping with synovitis, acne, pustulosis, hyperostosis, and osteitis.^27,58,59^ Syndromic HS has also been reported in association with genetic disorders, such as Down syndrome, Dowling-Degos disease, and keratitis-ichthyosis-deafness syndrome.^58^ Genetic mutations reported in syndromic HS vary in comparison to familial and sporadic HS and have been reported in *GEB2, MEFV, NCSTN, NLRC4, NOD2, OTULIN, POFUT1, PSENEN, PSTPIP1,* and *WRD1* (Table 3).

**Table 3:** Genetic mutations reported in syndromic HS

Sex	Diagnosis	Gene	Change in DNA	Change in protein	Origin	Familial/sporadic	Reference
F	PASH	*GJB2*	c.35delG	p.(G12Vfs*2)	NR	Sporadic	Marzano et al., 2022^27^
F	PAPASH & FMF	*MEFV*	c.2080A>G	p.(M695V)	NR	Sporadic	Marzano et al., 2022^27^
F	PAPASH & FMF	*MEFV*	c.2177T>C	p.(V726A)	NR	Sporadic	Marzano et al., 2022^27^
M	PAPASH	*NCSTN*	c.1140_1141del	p.(D381Sfs*7)	NR	Sporadic	Marzano et al., 2022^27^
M	PASH/SAPHO	*NCSTN*	c.482delA	p.(1162Yfs*57)	NR	Sporadic	Marzano et al., 2022^27^
M	SAPHO	*NCSTN*	c.278delC	p.P93LfsX15	Han Chinese	Sporadic	C. Li et al., 2018^59^
Family	PASH	*NCSTN*	c.1635C>G	p.Tyr545*	Iranian	Familial	Faraji Zonooz et al., 2016^60^
NR	PASH	*NCSTN*	c.344_351del	p.Thr115Asn*20	NR	Unknown (adopted child)	Duchatelet et al., 2015^61^
M	PASH/SAPHO	*NLRC4*	c.2668T>C	p.(C890R)	NR	Sporadic	Marzano et al., 2022^27^
F	PAPASH	*NLRC4*	c.541C>T	p.(R181X)	NR	Sporadic	Marzano et al., 2022^27^
M	PASH	*NOD2*	c.2104C>T	p.(R702W)	NR	Sporadic	Marzano et al., 2022^27^
M	PASH	*NOD2*	c.3017dupC	p.(L1007Pfs*2)	NR	Sporadic	Marzano et al., 2022^27^
F	PASH	*NOD2*	c.2722G>C	p.(G908R)	NR	Sporadic	Marzano et al., 2022^27^
M	PASH	*OTULIN*	c.209T>C	p.(I70T)	NR	Sporadic	Marzano et al., 2022^27^
F	PASH	*OTULIN*	c.345G>T	p.(Q115H)	NR	Sporadic	Marzano et al., 2022^27^
Family	HS/DDD	*POFUT1*	c.891G>A	p.Trp297*	NR	Familial	García-Gil et al., 2021^62^
M	HS/DDD	*PSENEN*	c.62_1G>T	NR	NR	Familial	Peter et al., 2021^63^
Family	HS/DDD	*PSENEN*	c.66delG	p.F23LfsX46	NR	Familial	Xiao et al., 2020^64^
F	PAPASH & FMF	*PSTPIP1*	c.831G>T	p.(E277D)	NR	Sporadic	Marzano et al., 2022^27^
Family	PASH	*PSTPIP1*	c.1034A>G	p.Y345C	Japanese	Familial	Saito et al., 2018^65^
M	PASH	*PSTPIP1*	c.1213C>T	p.Arg405Cys	NR	NR	Calderón-Castrat et al., 2016^66^
M	PASH/SAPHO	*WRD1*	c.323A>G	p.(H108R)	NR	Sporadic	Marzano et al., 2022^27^

DDD, Dowling-Degos disease; HS, hidradenitis suppurativa; NR, not reported; PAPASH, pyodermic arthritis, pyoderma gangrenosum, acne, and suppurative hidradenitis; PASH, pyoderma gangrenosum, acne, and suppurative hidradenitis; SAPHO, pyoderma gangrenosum, acne, and suppurative hidradenitis overlapping with synovitis, acne, pustulosis, hyperostosis, and osteitis.

In the study of a multigenerational Iranian family, whole genome linkage analysis and whole-exome sequencing (WES) revealed 2 rare heterozygous variants, one in *NCSTN* and one in *TTC24;* however, only the *NCSTN* mutation was found to be causative in the subjects with PASH.^60^ In 2 separate case studies of HS and Dowling-Degos disease, a rare autosomal dominant disease characterized by reticulate hyperpigmentation and dark brown papules, mutations in *PSENEN* were discovered by Sanger sequencing, while another case study reported a mutation in *POFUT1.*^62–64^ Marzano et al.^27^ utilized WES to identify mutations in 10 syndromic HS patients, rather than the targeted panels used in the case studies of the familial and sporadic case studies, and yielded a variety of genes within and outside of the GSC. Genetic variants were discovered in *MEVF, PSTPIP1, NLRC4, WDR1, NOD2, OTULIN, NCSTN,* and *GJB2*, which may be attributed to identification by WES, the presence of additional inflammatory disorders, or the polygenic nature of HS.^27^ The variety of mutations seen in cases of syndromic HS is indicative of the complex, multifactorial nature of the disease pathogenesis.

## Discussion

This review demonstrates that more research is needed to better understand the role genetics plays in HS. Approximately 30% of individuals diagnosed with HS report having a positive family history; however, very few studies demonstrate monogenic familial transmission of HS.^11,55^ Pink et al. evaluated mutations in the GSC and found that mutations in the genes that make up the GSC occur in less than 7% of HS cases and often have a more severe phenotype and earlier age of onset.^21,55^ Additional studies conducted by Liu et al.^48^ and Vural et al.^21^ indicated that the incidence of GSC mutations may be even less frequent, especially in sporadic cases. The case studies of syndromic HS reported a variety of genetic mutations associated with HS, some of which were familial, while others were sporadic, suggesting that other pathways may be involved in the pathogenesis of HS and other potential mutations that have yet to be evaluated.

This study was limited to the case studies available in PubMed, the majority of which used targeted gene panels to detect genetic mutations. WES and genome-wide association studies may provide greater insight into genetic information that may be linked to the pathogenesis of HS. Other risk factors for HS include obesity and smoking; therefore, epigenetic factors should also be taken into consideration.^11,23^ Additionally, evaluating familial, sporadic, and syndromic HS individually as distinct subsets of HS may allow for a better understanding of the pathogenesis in each form of the disorder.

## Conclusion

Further work is needed to evaluate distinct phenotypes amongst HS subtypes as well as genotype-phenotype correlation. Multiomics tools may offer a more integrated approach to evaluating the complex pathogenesis of HS. Combining information on the genome, transcriptome, and proteome will allow for a more comprehensive look into the pathogenesis of HS, while potentially opening the doors for better diagnostic tools as well as more effective therapeutics.

## Conflicts of interest

S.M.E. is an employee of Bristol Myers Squibb, Princeton, NJ. When the article was written, B.L. was also an employee of Bristol Myers Squibb. The other authors have no conflicts of interest to disclose.

## Funding

None.

## Study approval

N/A

## Author contributions

All authors participated sufficiently in the intellectual content, the analysis of data, and/or the writing and reviewing of the manuscript to take public responsibility for it.
